# Ascitic Fluid Lactate Level as a Predictor of Mortality in Cirrhotic Patients Having Spontaneous Bacterial Peritonitis (SBP)

**DOI:** 10.7759/cureus.53243

**Published:** 2024-01-30

**Authors:** Danish Kumar, Raja Taha Yaseen, Muhammad qaiser Panezai, Muhammad Usman Naeem, Hina Ismail, Zain Majid, Nasir Mehmood, Muhammad Ali Khalid, Ghulamullah Lail, Nasir Hassan Luck

**Affiliations:** 1 Department of Hepatogastroenterology, Sindh Institute of Urology and Transplantation, Karachi, PAK; 2 Department of Gastroenterology, Sheik Zayed Hospital, Lahore, PAK; 3 Department of Hepatology, Sheik Zayed Hospital, Lahore, PAK; 4 Department of Hepatogastroenterology, Sindh Institute of Urology and Transplantation, KARACHI, PAK; 5 Department of Medicine: Gastroenterology, Jinnah Medical & Dental College, Karachi, PAK

**Keywords:** cirrhosis, sbp, mortality, lactate, ascitic fluid

## Abstract

Introduction

Limited studies are available for predicting mortality in patients with spontaneous bacterial peritonitis (SBP) based on ascitic fluid analysis. Recently, a proposition has been made regarding the role of ascitic fluid lactate as a better prognostic indicator of mortality in cirrhotic patients with SBP. Therefore, we aimed to evaluate the utility of ascitic fluid lactate in predicting mortality in cirrhotic patients with SBP.

Methods

This was a prospective, observational study that was conducted in the Hepato-Gastroenterology Department of Sindh Institute of Urology and Transplantation (SIUT), Karachi from 1 January 2022 to 31 December 2022. All the patients having liver cirrhosis with ascites, aged between 18 and 65 years, and presenting with fever and/or abdominal pain were recruited in the study in the first six months (i.e., from 1 January 2022 to 30 June 2022) and were followed for six more months for the outcome. However, those patients on dialysis or those with hepatocellular carcinoma, any other malignancy as per a history of solid organ transplant, a history of HIV infection, or those underlying systemic sepsis or infections other than SBP were excluded from the study. The presence or absence of SBP was confirmed by doing the ascitic fluid analysis. Ascitic fluid lactate levels were also requested in each patient. Mortality was assessed at one, two, three, and six months, respectively. All the data were analyzed using SPSS version 23.0. The area under the receiver operating curve (AUROC) was obtained for ascitic fluid lactate for predicting mortality in SBP. At an optimal cutoff, the diagnostic accuracy of ascitic fluid lactate was obtained.

Results

The total number of cirrhotic patients included in the study was 123. The majority of the patients belong to Child Turcotte Pugh (CTP) class C (n = 88; 71%). Two third of the patients (65.8%; n = 81) had viral hepatitis i.e., hepatitis B, D, and/or C, as the cause of cirrhosis. Overall mortality was observed in 51(41.5%) patients. Ascitic fluid lactate was significantly raised in patients with SBP than in patients with non-SBP (p = 0.004). The AUROC of ascitic fluid lactate was highest at three months (AUROC = 0.88) followed by six months (AUROC = 0.84), two months (AUROC = 0.804), and one month (AUROC=0.773). At an optimal cut-off of more than or equal to 22.4 mg/dl, ascitic fluid lactate had a sensitivity of 84.9%, specificity of 85.7%, positive predictive value (PPV) of 97.3%, negative predictive value of 42.8% with diagnostic accuracy of 85% in predicting overall mortality in patients with SBP. On sub-analysis, the diagnostic accuracy of ascitic fluid lactate was highest at six months followed by at three, two, and one month, respectively.

Conclusion

Ascitic fluid lactate showed a good diagnostic utility in predicting the overall mortality in patients with SBP with the best diagnostic accuracy in predicting long-term (six months) mortality. However, further studies are required to validate our results.

## Introduction

Ascites is termed as the accumulation of fluid in the peritoneal cavity, which can be clinically detectable when it is more than 1.5 liters [1]. Various etiological factors are linked to the presence of ascites, including cirrhosis, heart failure, renal disease, malignancies, tuberculosis, or pancreatic disease [2]. Portal hypertension in cirrhotic patients leads to the development of ascites [3]. Being immunocompromised, cirrhotic patients are prone to develop infections, which leads to multiorgan failure thus associated with high mortality [4]. Spontaneous bacterial peritonitis (SBP), an ascitic fluid infection, characterized by ascitic polymorphonuclear cell count ≥ 250/µL, is among the commonest of infections in decompensated chronic liver disease with more than 50% mortality at one year [5]. Piano et al. reported a 27% prevalence of SBP (n = 1302) in liver cirrhosis patients [6]. Ding et al. concluded that gram-negative bacteria, in particular E. coli, is the major culprit pathogen causing SBP in their study population [7].

 Moreover, SBP is associated with higher morbidity and mortality [8]. Devani et al. reported longer hospital stays and a higher risk of acute kidney injury in cirrhotic patients with SBP as compared to patients without SBP [9]. A meta-analysis reported a high mortality of 30.3% and 63% in patients with SBP at one month and one year, respectively [5]. A large retrospective study conducted in Israel reported increased rates of long-term mortality in cirrhotic patients with SBP approaching approximately 90% [10].

Studies have reported variable biochemical parameters to diagnose SBP [11]. However, there are limited studies to predict mortality in SBP based on ascitic fluid analysis. Simbrunner et al. found increased mortality among patients with cirrhosis having ascitic fluid polymorphonuclear cell (PMN) count between 125 and 250/µL or more than 250/uL [12]. Recently, Mani et al., in a single study, proposed the role of ascitic fluid lactate as a better prognostic indicator of mortality in cirrhotic patients with SBP with sensitivity, specificity, positive predictive value, and negative predictive value of 100%, 73.3%, 80.5%, and 100%, respectively, at 90 days [13]. The author also proposed ascitic lactate as a better prognostic marker as compared to the model for end-stage liver disease (MELD), neutrophil-to-lymphocyte ratio (NLR), and arterial lactate.

Lactate is produced by anaerobic glycolysis and increased during stress, hypoxia, and sepsis [14]. Serum lactate and lactate clearance have been utilized for the prediction of mortality in critically ill patients who are admitted to an intensive care unit [15]. In another study, Gao et al. also stated similar results regarding lactate clearance as an independent predictor of mortality in critically ill cirrhotic patients [16].

As studies have documented serum lactate as a predictor of short-term mortality in cirrhotic patients and Mani et al. proposed ascitic fluid lactate as a prognostic marker in cirrhotic patients with SBP, this study was mainly performed in alcoholics while viral hepatitis is the most common etiology of cirrhosis in our population [13]. Therefore, we aimed to evaluate the utility of ascitic fluid lactate in predicting mortality in cirrhotic patients with SBP. The result of this study would help us identify patients with a higher risk of mortality in SBP.

## Materials and methods

Study methodology

After approval from the Ethical Review Committee, Sindh Institute of Urology and Transplantation (ERC-SIUT) (Approval no.440), this prospective, observational study was conducted at the Department of Hepato-Gastroenterology, Sindh Institute of Urology and Transplantation (SIUT), Karachi over a period of one year, i.e., 1 January 2022 to 31 December 2022. For the first six months, patients fulfilling the inclusion criteria were included in the study and were followed for the next six months for the outcome.

All patients with liver cirrhosis as per the operational definition and aged between 18 and 65 years, presenting with fever and/or abdominal pain were included in the study (Table 1). However, the patients on dialysis or acute kidney injury(AKI) at presentation, those with a history of hepatocellular carcinoma, any other malignancy as per history, prior history of solid organ transplantation, history of infection with HIV, or those with underlying systemic sepsis or infections other than SBP were excluded from the study.

**Table 1:** Operational definition of cirrhosis of the liver, SBP, and MELD-Na SBP: spontaneous bacterial peritonitis; MELD-Na: model for end-stage liver disease-sodium

Operational Definitions:
Cirrhosis of the liver [17]: Patients with liver cirrhosis were identified on the basis of ultrasound abdomen findings. The presence of three of the following features along with ascites was considered as cirrhosis of the liver: 1. Altered echotexture of the liver; 2. Irregular liver margins; 3. Spleen size more than 12 cm; 4. Portal vein diameter of more than 12 mm.
Spontaneous bacterial peritonitis (SBP) [18]: SBP was labeled in cirrhotic patients with ascites having ascitic fluid polymorphonuclear leukocyte count ≥250/µL.
Model for end-stage liver disease-sodium (MELD-Na) [19]: MELD-Na is similar to MELD but it includes serum sodium. It was calculated by the MDCalc calculator: MELD-Na = MELD Score - Na - 0.025 x MELD x (140-Na) + 140

Sampling technique and sample size

Patients were enrolled by non-probable consecutive sampling. The total population of cirrhotic patients with and without SBP comprised 123 patients during a six-month period based on the previous estimate of SBP in cirrhotic patients with a margin of error of 3% and a 95% confidence interval [13].

Data collection procedure

Patients meeting the inclusion criteria were recruited from 1 January 2022 to 30 June 2022 and were then followed for six months for the outcome (i.e., 1 July 2022 to 31 December 2022). All the patients with decompensated chronic liver disease admitted to a ward or intensive care unit (ICU) were included after taking the informed written consent. Demographic data (age, gender), cause of liver disease, and laboratory parameters (complete blood count (CBC), plasma lactate, liver function test (LFTs), urea, creatinine, electrolytes (UCE), serum albumin, international normalized ratio (INR), blood culture) were obtained at the time of admission. Ultrasound abdomen-guided ascitic fluid paracentesis was done via a 20 cc syringe from the left lower quadrant, 3 cm cephalad, and 1-2 cm medial to the anterior superior iliac spine with a 22G needle after all aseptic measures [20]. Ascitic fluid (3 cc) was collected in a red-topped bottle for ascitic fluid protein and albumin. Further, 3 cc ascitic fluid was added to each purple-topped bottle containing ethylenediaminetetraacetic acid (EDTA) for total leucocyte count (TLC) and neutrophil count, and in a gray-topped bottle containing sodium fluoride for ascitic fluid lactate. Ten ccs of ascitic fluid were inoculated into blood culture bottles for aerobic and anaerobic bacterial growth at the bedside. All ascitic fluid sampling was done at the bedside. Samples were then sent to the laboratory within 15 minutes of collection.

Patients having SBP and non-SBP were followed up during the period of hospitalization and after discharge at the outpatient department or by telephonic communication with patients or relatives monthly for the initial three-month period and then at six months. Mortality was assessed at one, two, three, and six months, respectively.

Statistical analysis

Data were entered and analyzed using Statistical Package for the Social Sciences (SPSS) version 23 (IBM Corp., Armonk, NY, USA). Continuous variables were presented as mean ± standard deviation while categorical variables were expressed as frequencies and percentages. Continuous variables were analyzed and compared using the student's T-test while categorical variables were analyzed and compared using the chi-square test.

The area under the Receiver operating characteristic (AUROC) curves were computed for the ascitic fluid lactate in predicting one, two, three, and six-month mortality. At optimal cutoff levels of ascetic fluid lactate, the sensitivity, specificity, positive predictive value (PPV), negative predictive value (NPV), and diagnostic accuracy were calculated. A p-value of ≤ 0.05 was considered statistically significant.

## Results

The total number of cirrhotic patients included in the study was 123. Around two-thirds of the patients were males (n=85; 69.1%). The mean age was 45.1 ± 15.2 years. The majority of the patients belonged to Child Turcotte Pugh (CTP) class C (n = 88; 71%). Two-thirds of the patients (65.8%; n = 81) had viral hepatitis, i.e., hepatitis B, D, and/or C, as the cause of cirrhosis followed by autoimmune hepatitis (n = 16), alcoholic liver disease, non-alcoholic steatohepatitis (NASH), Wilson disease, and other etiologies. SBP was diagnosed in 60 (48.7%) patients at the time of admission. Mean ascitic fluid TLC and lactate were 1414 ± 4406 mm^3^ and 30.4 ± 20.1 mg/dl, respectively. At the end of six months, overall 72 (58.5%) patients survived while mortality was noted in 51 (41.5%) patients. The mortality noted in SBP at one, two, three, and six months was 36.7%, 45%, 50%, and 55% respectively. Baseline characteristics of the patients are mentioned in Table 2.

**Table 2:** Baseline characteristics of the population included in the study (n-123) The data were expressed as N (%) for categorical variables and mean±SD for continuous variables. CTP: Child Turcotte Pugh; HBV: Hepatitis B virus; HCV: Hepatitis C virus; HDV: Hepatitis D virus; AIH: autoimmune hepatitis; NASH: non-alcoholic steatohepatitis; SBP: spontaneous bacterial peritonitis; MELD-Na: model for end-stage liver disease-sodium; TLC: total leucocyte count

Study population			N (%)
Gender		Male	85 (69.1)
		Female	38 (30.9)
Child Turcotte Pugh (CTP)		B	35 (28.5)
		C	88 (71.5)
Cause of cirrhosis	HBV		17 (13.8)
	HCV		44 (35.8)
	HBV/HCV		6 (4.9)
	HBV/HDV		12 (9.8)
	HBV/HCV/HDV		2 (1.6)
	AIH		16 (13)
	Cryptogenic cirrhosis		12 (9.7)
	Alcoholic liver disease		4 (3.3)
	NASH		5 (4.1)
	Wilson Disease		5 (4.1)
SBP	Yes		60 (48.8)
	No		63 (51.2)
Overall Mortality			51 (41.5)
One month			34 (27.6)
Two months			41 (33.3)
Three months			47 (38.2)
Six months			51 (41.5)
Mortality in SBP			33(55)
One month mortality in SBP			22(36.7)
Two months mortality in SBP			27(45)
Three months mortality in SBP			30(50)
Six months mortality in SBP			33(53)
Mean age (years ± S.D)			45.1 ± 15.2
Hemoglobin (g/dL)			9.6 ± 1.9
Total Leucocyte Count (x10^9^/L)			8.9 ± 5
Platelet (x10^9^/L)			118 ± 81
Segmented neutrophil fraction (%)			74.3 ± 11.6
Lymphocyte fraction (%)			14.4 ± 19.2
Serum Creatinine (mg/dl)			1.3 ± 1.0
Total Bilirubin (mg/dL)			6.5 ± 7.5
Aspartate Transaminase (AST) (U/L)			120 ± 102
Alanine Transaminase (ALT) (U/L)			63 ± 63.4
Serum Albumin (g/dL)			2.3 ± 0.5
International Normalized Ratio (INR)			1.6 ± 0.6
MELD-Na			23.1 ± 7.5
Ascitic TLC (mm^3^)			1414 ± 4406
Ascitic Lactate (mg/dL)			30.4 ± 20.1

On comparison of patients between the SBP and non-SBP groups, patients in the SBP group had higher significant overall mortality as compared to the non-SBP group over a 180-day duration (p = 0.003). International normalized ratio (INR), ascitic TLC, and ascitic neutrophils were significantly higher in the former group. Ascitic fluid lactate was significantly raised in patients with SBP than in patients with non-SBP (p = 0.004) (Table 3).

**Table 3:** Comparison of baseline variables in terms of SBP (n-123) The data were represented as N (%) for categorical variables and Mean±SD for continuous variables. A p-value of <0.05 was considered statistically significant. SBP: spontaneous bacterial peritonitis; CTP: Child Turcotte Pugh; TLC: total leucocyte count

Variable		Spontaneous Bacterial Peritonitis		p-value
		Present (n-60) N (%)	Absent (n-63) N (%)	
Gender	Males	38 (63.3)	47 (74.6)	0.176
	Females	22 (36.7)	16 (25.4)	
CTP class	B	16 (26.6)	19 (30.1)	0.668
	C	44 (73.4)	44 (69.9)	
Mortality	Yes	33 (55)	18 (28.6)	0.003
	No	27 (45)	45 (71.4)	
Age (Years)		44.8 ± 16.1	45.5 ± 14.4	0.797
Hemoglobin (g/dL)		9.4 ± 2.0	9.8 ± 1.9	0.243
Total leucocyte count (x10^9^/L)		9.7 ± 5.6	8.3 ± 4.2	0.126
Platelet Count (x10^9^/L)		105 ± 62	131 ± 94	0.071
Segmented neutrophil fraction (%)		76.3 ± 10.3	72.5 ± 12.6	0.067
Lymphocyte fraction (%)		12.5 ± 7.6	15.5 ± 10.3	0.063
Serum creatinine (mg/Dl)		1.5 ± 1.3	1.2 ± 0.7	0.150
Serum Na (mEq/L)		132 ± 6.8	130 ± 5.8	0.209
Total bilirubin (mg/dl)		6.8 ± 7.6	6.3 ± 7.5	0.711
Aspartate transaminase (AST) (U/L)		122 ± 109	117 ± 97	0.746
Alanine transaminase (ALT) (U/L)		57 ± 39	68 ± 81	0.333
Serum albumin (g/dL)		2.3 ± 0.5	2.4 ± 0.54	0.087
International normalized ratio (INR)		1.8 ± 0.6	1.5 ± 0.49	0.015
Ascitic TLC (mm^3^)		2749 ± 6050	143 ± 103	0.001
Ascitic neutrophils (mm^3^)		2599 ± 6081	56 ± 46	0.001
Ascitic lactate (mg/dL)		36.5 ± 24.5	24.5 ± 20.3	0.004

**Figure 1:**
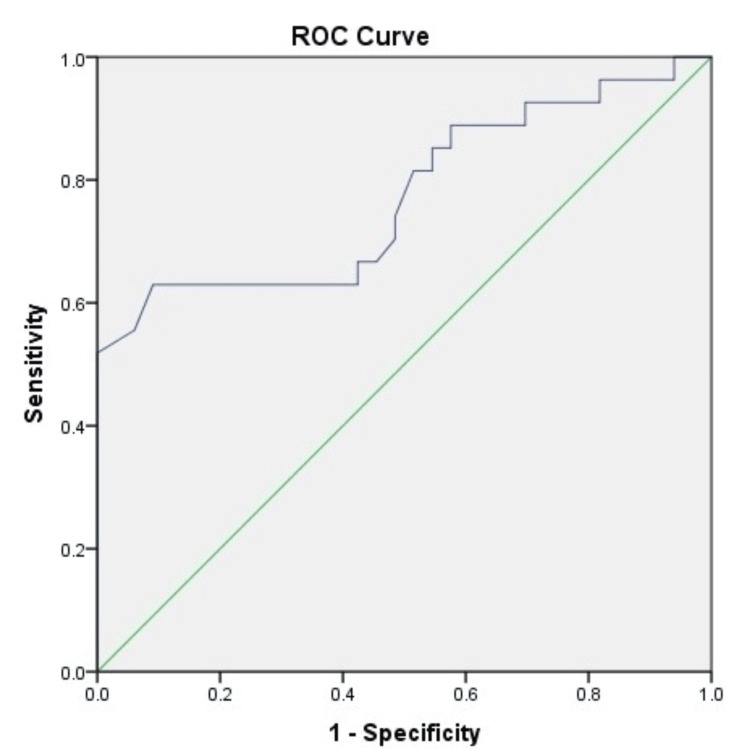
AUROC for ascitic fluid lactate in predicting one mortality in patients with SBP (AUROC-0.773, p-value<0.001) SBP: spontaneous bacterial peritonitis; AUROC: area under the receiver operating characteristic

**Figure 2:**
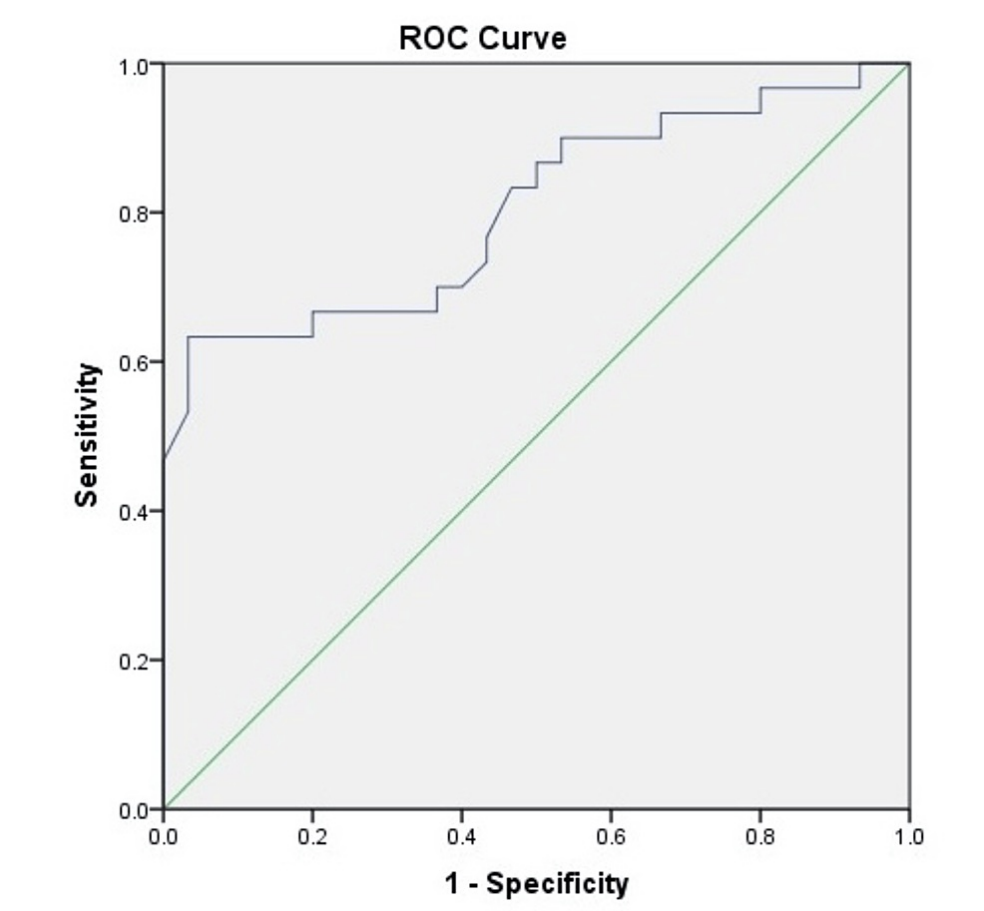
AUROC for ascitic fluid lactate in predicting two months mortality in patients with SBP (AUROC: 0.804, p-value<0.001) SBP: spontaneous bacterial peritonitis; AUROC: area under the receiver operating characteristic

**Figure 3:**
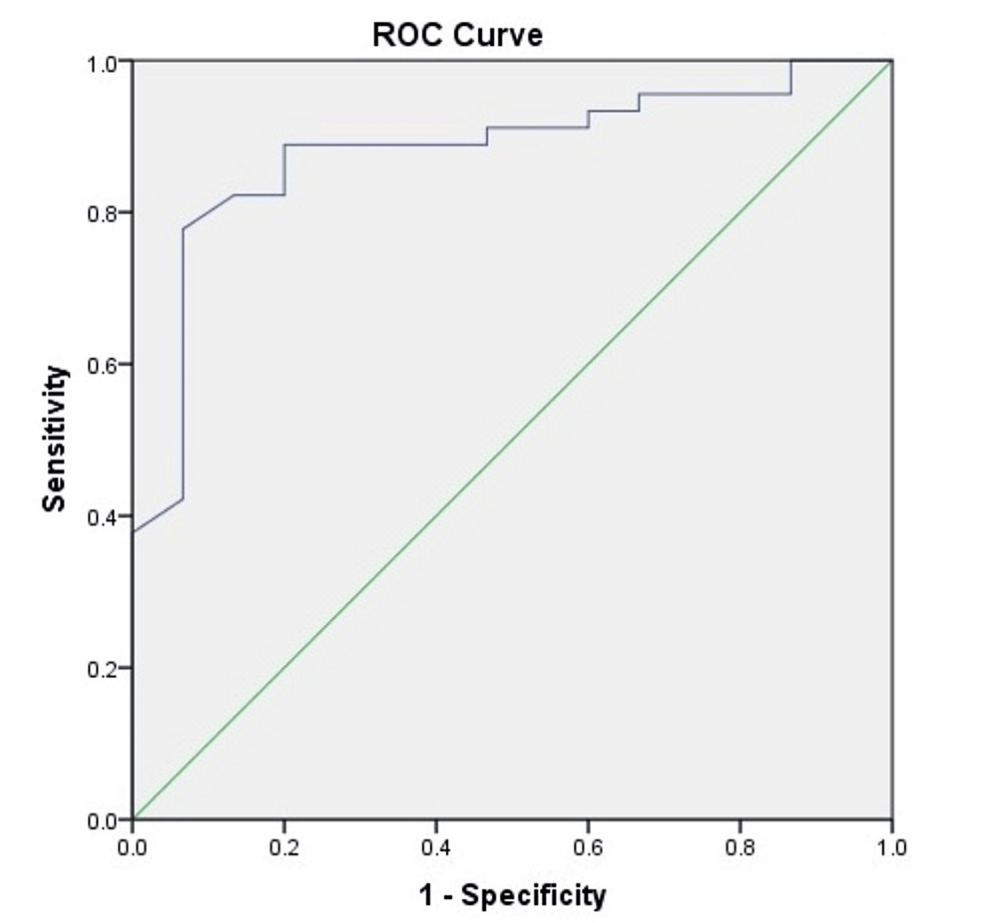
AUROC for ascitic fluid lactate in predicting three months mortality in patients with SBP (AUROC-0.88, p-value<0.001) SBP: spontaneous bacterial peritonitis; AUROC: area under the receiver operating characteristic

**Figure 4:**
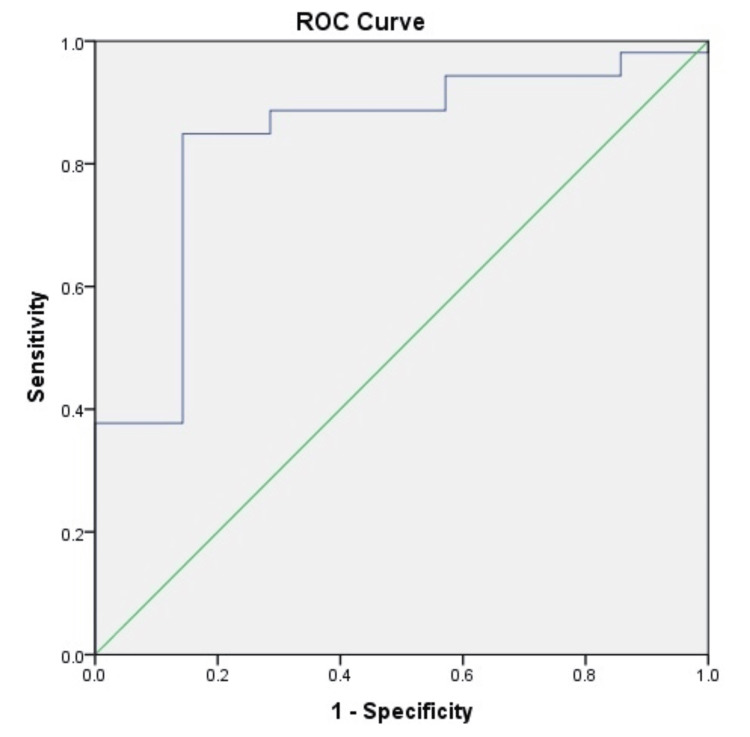
AUROC for ascitic fluid lactate in predicting six months mortality in patients with SBP (AUROC-0.84, p-value<0.004) SBP: spontaneous bacterial peritonitis; AUROC: area under the receiver operating characteristic

The AUROC analysis was computed for ascitic fluid lactate in predicting mortality in patients with SBP at one month, two months, three months, and six months (Figures 1-4). The AUROC of ascitic fluid lactate was highest at three months (AUROC = 0.88) (Figure 3) followed by six months (AUROC = 0.84), two months (AUROC = 0.804), and one month (AUROC = 0.773).

At a cut-off point of 22.4 mg/dl, ascitic fluid lactate had a sensitivity of 84.9%, specificity of 85.7%, positive predictive value (PPV) of 97.3%, and negative predictive value of 42.8% with diagnostic accuracy of 85% in predicting overall mortality in patients with SBP (Table 4). Thus, ascitic fluid lactate was effective and accurate in predicting overall (six months) mortality in SBP patients as compared to its role in predicting one-month mortality in patients with SBP.

**Table 4:** Performance of ascitic fluid lactate in predicting mortality in patients with SBP SBP: spontaneous bacterial peritonitis; AUROC: area under the receiver operating characteristic

Ascitic fluid lactate >22.4 mg/dl	Overall mortality	One-month mortality	Two-month mortality	Three-month mortality	Six-month mortality
Sensitivity	84.9%	87.80%	86.96%	77.92%	84.9%
Specificity	85.71%	60.98%	63.64%	82.61%	85.71%
Positive predictive value	97.83%	52.94%	58.82%	88.24%	97.83%
Negative predictive value	42.86%	90.91%	89.1%	69.1%	42.86%
Diagnostic accuracy	85%	69.92%	72.4%	79.67%	85%

## Discussion

Spontaneous bacterial peritonitis (SBP) is a common infection among cirrhotic patients [21]. Two-thirds of the patients had viral hepatitis as the cause of cirrhosis in our study, reflecting the prevalence of viral hepatitis in developing countries like Pakistan, which has the second-highest number of patients with hepatitis C globally [22]. Arvanti et al. described that mortality in patients with SBP was 30% at one month and around 60% at one year [5]. In our study, more than half of the patients having SBP (55%) died within six months, reflecting the same rate of mortality compared to other studies in the literature.

Previously, multiple studies have been performed to evaluate the factors that can predict mortality in patients with SBP but ascitic fluid lactate was first used by Mani et al. in predicting mortality [13]. In our study, we found that ascitic fluid lactate at a cut-off of more than or equal to 22.4 mg/dl was better in predicting the mortality in patients with SBP; these results were in concordance with the study previously done by Mani and his colleagues [13]. Although the majority of the patients in their study had alcoholic cirrhosis, the majority of the studied population had viral hepatitis as the cause of cirrhosis.

This shows that ascitic fluid lactate is increased in SBP regardless of the etiology of cirrhosis and carries a prognostic role in predicting mortality. On a comparative analysis with the previous studies, the AUROC for ascitic fluid lactate in predicting mortality in patients with SBP in our population was highest at six months, which was similar to that shown by Mani et al. [13].

In one of the recent studies, it was found that MELD and MELD-Na had limited ability to predict 90-day mortality in patients with SBP [23]. These results can be attributed to low sample sizes in the respective studies. However, in our study, we found that MELD-Na was higher in patients with SBP than non-SBP. In another study, they found MELD as an independent predictor of in-hospital mortality in SBP patients along with hepatic encephalopathy and ascitic prostaglandin E2 (PGE2) [24].

One study done by Sandhya et al. utilized the ascitic fluid lactate dehydrogenase (LDH) in the diagnosis of SBP and found that patients with ascitic fluid LDH less than 127.5 IU/L have fewer chances of having SBP but they evaluated its prognostic value in predicting mortality in patients with SBP [25]. Since LDH is raised in conditions involving inflammation or infection, it can be utilized in the future in predicting mortality in patients with SBP and compared with ascitic fluid lactate.

Hence, cirrhotic patients with fever and ascites should undergo thorough investigations to rule out SBP. However, a delay in reporting ascitic fluid total leukocyte count (TLC) and differential leukocyte count (DLC) is a hurdle in prompt treatment but ascitic fluid lactate can potentially guide regarding prompt treatment of patients.

Certain limitations can be attributed to our study. The first and foremost was the small sample size of this study. Second, it was a single-centered study. Last, various etiologies of cirrhosis were included in the study with the majority being viral hepatitis as the cause, so results could not be generalized to all the etiologies of cirrhosis.

However, one of the major strengths of this study is that it is the pioneer study showing the utility of ascitic fluid lactate as a predictor of mortality in SBP patients in the Asian population.

## Conclusions

The ascitic fluid lactate levels of >22.4 mg/dl showed excellent accuracy in predicting the overall mortality in patients with SBP with the best diagnostic accuracy in predicting mortality within the first six months. However, studies are required regarding the comparative analysis of the ascitic fluid lactate levels with other prognostic scores such as CTP and MELD scores in predicting mortality in this subset of patients. Therefore, future studies focussing on multi-centric studies with large sample sizes along with equal distribution of patients having various etiologies of cirrhosis should be undertaken for further validation of our results.
